# Endovascular recanalization of chronic total occlusion of the subclavian artery: fifteen years of technological evolution

**DOI:** 10.1186/s42155-026-00793-6

**Published:** 2026-10-27

**Authors:** Srđan Babić, Aleksandra Milačić, Vladimir Kovačević, Slobodan Pešić, Vladimir Mihajlović, Želimir Antonić, Milovan Bojić

**Affiliations:** 1 National Institute for Heart and Blood Vessels “Dedinje”, Belgrade, Serbia; 2 Faculty of Medicine, University of Belgrade, Belgrade, Serbia

**Keywords:** Subclavian artery, Chronic total occlusion, Endovascular therapy, Angioplasty, Stenting, Guidewire, Computed tomography angiography, Technological evolution

## Abstract

Fifteen years ago, endovascular recanalization of chronic total occlusion of the subclavian artery relied largely on coronary guidewires and conventional catheters. An early series achieved technical success in 46 of 56 patients (82.1%). Today, dedicated crossing wires, support catheters, improved imaging, and a wider choice of stents provide additional options for complex lesions. Yet published series report variable technical success and patency, and differences in patient selection and follow-up limit comparisons between treatment eras. This review revisits earlier techniques, examines what has changed in practice, and discusses persistent challenges, particularly heavily calcified and ostial occlusions near the vertebral artery. Technological progress has broadened treatment options, but its independent contribution to long-term outcomes remains uncertain.

## Introduction

Chronic total occlusion (CTO) of the subclavian artery (SA) represents one of the most technically challenging lesions in peripheral endovascular intervention. Although less common than aortoiliac or femoropopliteal occlusive disease, symptomatic SA CTO may result in vertebrobasilar insufficiency, upper extremity ischemia, coronary-subclavian steal syndrome, and compromised inflow through the left internal mammary artery in patients undergoing or following coronary artery bypass grafting. In appropriately selected symptomatic patients, revascularization is undertaken to relieve ischemia or steal-related symptoms and, when relevant, to preserve mammary graft inflow. Surgical reconstruction was historically considered the gold standard; however, during the last two decades, endovascular treatment (EVT) is commonly considered first in appropriately selected symptomatic patients, although CTO-specific comparative evidence is limited and surgical reconstruction remains relevant when EVT is unsuitable or fails [1–5].

An earlier clinical report, published online in March 2011 and in the 2012 journal issue, presented one of the earliest dedicated long-term clinical series evaluating EVT of CTO of the SA [1]. In that study, successful recanalization was achieved in 82.1% of patients, with primary patency rates of 97.9% and 82.7% after 1 and 3 years, respectively. At a time when dedicated peripheral CTO devices were not available, the procedure relied primarily on coronary guidewires, standard guiding catheters, balloon angioplasty, and first-generation balloon-expandable or self-expanding stents. Importantly, that report proposed that future improvements in technical success would likely depend on the development of dedicated guidewires specifically designed for CTO crossing and on more accurate preprocedural imaging for optimal lesion characterization and procedural planning [1].

Many of these tools are now available, although their individual effects on CTO outcomes remain uncertain. Contemporary EVT has evolved through the introduction of dedicated CTO guidewires, microcatheters, continued use and refinement of bidirectional crossing strategies, low-profile delivery systems, advanced balloon-expandable and self-expanding stents, selective use of covered stents, high-resolution computed tomography angiography (CTA), and adjunctive intravascular imaging [5–7]. These developments have broadened the available procedural options, particularly during assessment and crossing of complex lesions.

Against this background, this review revisits the original clinical series and examines how lesion assessment and recanalization techniques have evolved, while comparing reported technical success, long-term patency, and the challenges that remain.

## Endovascular treatment in 2012: the starting point

When the earlier clinical series was published in 2012, EVT for SA CTO was still considered technically demanding and was performed in only a limited number of specialized vascular centers [1]. Most published reports had included predominantly stenotic lesions, whereas dedicated studies focusing exclusively on complete SA occlusions were scarce and generally involved small patient cohorts with limited follow-up [2–4, 6]. In that series, the preprocedural imaging strategy evolved over the study period. Between 1999 and 2005, digital subtraction angiography (DSA) was used in 15 patients, whereas multislice CTA was introduced in 2005 and subsequently used in 41 patients. Imaging assessment focused on occlusion length, calcification, and the relationship to the vertebral artery origin. Thus, CTA was already part of preprocedural planning, although contemporary multiplanar reconstructions allow more detailed assessment of the proximal cap and vessel course [1].

Crossing the occlusion was the main technical challenge. In the absence of dedicated peripheral CTO devices, recanalization was performed with conventional 0.014-inch coronary guidewires, coronary guiding catheters, and balloon catheters. After successful crossing, balloon predilatation allowed exchange to a 0.035-inch guidewire, followed by peripheral balloon angioplasty and stent implantation. Subintimal recanalization was deliberately avoided, with repeated attempts to achieve true intraluminal passage when necessary [1]. Combined femoral and upper-extremity access was used in 41 of 56 patients (73.2%) in that series. This approach provided additional guidewire support and allowed the occlusion to be approached from either direction when necessary. It remains useful in selected complex cases today [1]. Balloon-expandable stents were preferred for ostial lesions because of their precise deployment, whereas self-expanding stents were generally used for longer or more tortuous lesions. Stent selection continues to depend primarily on lesion anatomy, as comparative evidence specific to SA CTO remains limited [1, 6].

The advances in guidewire technology and preprocedural imaging anticipated in the earlier report are now available in contemporary practice. However, the choice of technique still depends on lesion anatomy, particularly its relationship to the vertebral artery, and procedural safety [1, 5, 6].

## Fifteen years of technological evolution

Advances in preprocedural imaging, guidewire technology, crossing strategies, stent design, and adjunctive imaging provide more detailed anatomical information and additional options for lesion crossing, although comparative outcome benefits have not been demonstrated. The essential procedural decisions, however, still concern lesion anatomy, safe wire passage, and appropriate stent placement [1, 6].

### Preprocedural imaging

Multidetector CTA permits more detailed assessment of the occlusion before intervention. It helps characterize lesion length, calcification, stump morphology, vessel tortuosity, vertebral artery origin, and distal vessel quality, which may inform patient selection and the crossing strategy. Whether routine CTA improves technical success, safety, or patency in subclavian CTO has not been established by comparative studies [5–7].

### Guidewires and crossing strategies

Later subclavian CTO series describe the selective use of dedicated crossing wires and support catheters [6]. Coronary CTO algorithms and microcatheter studies offer technical background, but not direct evidence of improved subclavian outcomes [8, 9]. Contemporary guidewires with varied tip loads, enhanced torque control, and hydrophilic or polymer-jacketed coatings allow tailored crossing strategies according to lesion morphology. Combined femoral and upper-extremity access can provide alternative crossing directions and additional wire support in selected complex occlusions; this strategy was already employed in the earlier series [1, 6, 10]. Despite these advances, technical success reflects lesion morphology, procedural strategy, and operator experience; comparative subclavian CTO data isolating individual device effects remain limited [1, 6, 11].

### Stent technology and adjunctive imaging

Modern balloon-expandable and self-expanding stents differ from earlier platforms in design and delivery characteristics, but comparative subclavian CTO outcome data are limited. In the series of Niu et al., a covered stent was used to manage dissection extending towards the aortic arch; in that case the left vertebral origin was covered and vertebral flow was affected [6]. This illustrates the value of a bailout option, but also its potential anatomical cost. Furthermore, intravascular ultrasound (IVUS) may assist vessel sizing and evaluation of stent expansion, but the available observational evidence includes predominantly stenotic lesions and does not establish a CTO-specific clinical benefit [12]. Distal embolic protection has been described in a four-patient series of chronic brachiocephalic and subclavian occlusions, with three successful recanalizations. This experience demonstrates procedural feasibility but cannot establish a reduction in cerebral embolic events [13].

The primary technical benefits and clinical roles of these individual technological advances are outlined in Table 1. The major procedural components and their evolution over the 15-year period are summarized in Table 2.

**Table 1:** Major technological advances and their potential clinical roles

Technological advance	Main technical benefit	Practical interpretation	Current role
Multidetector CTA	Accurate anatomy	Improved planning	As indicated by lesion complexity
Dedicated CTO guidewires	Higher penetration and torque	Potential crossing aid; subclavian CTO outcome benefit unproven	Selected lesions
Microcatheters	Wire support and exchange	Wire support and exchange; comparative benefit unproven	When additional wire support is needed
Bidirectional access	Better guidewire support	An option in complex CTO; already used historically	Selected complex lesions
Low-profile balloons	Improved deliverability	Better lesion preparation	As needed for lesion preparation
Contemporary stents	Lesion scaffolding and lumen restoration	No proven CTO-specific comparative superiority	Selected according to anatomy
Covered stents	Selected bailout for dissection or arterial injury	Case-level evidence only; routine benefit not established	Selective
IVUS	Accurate sizing	Potential adjunct; CTO-specific benefit unproven	Selective

*Abbreviations*: *CTA* computed tomography angiography, *CTO* chronic total occlusion, *IVUS* intravascular ultrasound

**Table 2:** Evolution of endovascular treatment of chronic total occlusion of the subclavian artery

Procedural component	Original series (Jan 1999–Feb 2010; published 2012)	Current practice
Preprocedural imaging	DSA (1999–2005; 15 patients); multislice CTA (from 2005; 41 patients)	CTA with multiplanar reconstruction; selective 3D rendering
Lesion characterization	Length, calcification, vertebral artery origin	Plaque morphology, stump configuration, calcium burden, tortuosity
Guidewire technology	Conventional 0.014-inch coronary guidewires (with step-up to 0.035 inch)	Dedicated peripheral CTO guidewires
Support devices	Coronary guiding catheters and over-the-wire balloon support	Microcatheters and support catheters
Crossing strategy	Combined femoral and upper-extremity access in 41/56 (73.2%)	Continued use and refinement of bidirectional approaches
Balloon technology	Conventional PTA balloons	Low-profile, high-pressure balloons
Stent technology	First-generation BE/SE stents	Contemporary BE, SE, and selective covered stents
Intraprocedural imaging	Angiographic guidance	Angiography, with selective adjunctive IVUS
Embolic protection	Not described as routine in the original 2012 series	Selective use; CTO-specific stroke reduction unproven

*Abbreviations*: *3D* three-dimensional, *BE* balloon-expandable, *CTA* computed tomography angiography, *CTO *chronic total occlusion, *DSA* digital subtraction angiography, *IVUS* intravascular ultrasound, *PTA* percutaneous transluminal angioplasty, *SE* self-expanding

## Have technological advances improved clinical outcomes?

The series summarized in Table 5 were selected because they report technical success together with patency or follow-up outcomes. CTO-focused cohorts are presented separately from mixed stenosis/occlusion series, and studies that also include innominate lesions are identified.

Published SA CTO series have reported variable technical success rates: 82.1% (46/56) in the original 2012 cohort, 77.6% (52/67) in Liu et al., 93.8% (15/16) in Çakar et al., and 91.3% (21/23) in Niu et al. [1, 6, 14, 15]. Differences in patient selection and lesion characteristics limit direct comparisons of technical success between these studies (Table 5).

Liu et al. also included a nonrandomized comparison with 21 patients treated by bypass surgery, in whom technical success was 100%. Three-year primary patency was 92.1% after successful EVT and 93.1% after surgical treatment. However, these results do not establish the superiority of either approach because treatment allocation was not randomized [14].

Technical success and long-term patency are distinct endpoints. Technical success refers to successful recanalization and treatment of the occlusion during the initial procedure, according to each study’s definition. Primary patency describes uninterrupted vessel patency without repeat intervention, whereas secondary patency refers to patency maintained or restored after additional treatment, according to the definitions used in each study.

Patency rates are generally reported for successfully treated lesions and should not be interpreted as overall treatment success among all attempted CTO cases. Studies including both stenoses and occlusions are presented separately in Table 5.

Contemporary CTO guidewires and support catheters have substantially expanded the technical options available for subclavian artery recanalization and may facilitate crossing of lesions that are difficult to treat with conventional equipment. However, the independent contribution of these technological advances to procedural success cannot be reliably quantified from the available heterogeneous clinical series [1, 5–7].

Lesion characteristics also play an important role in procedural planning. Long occlusions, severe circumferential calcification, absence of a proximal stump, marked vessel tortuosity, and involvement of the vertebral artery origin may increase crossing difficulty. Although these anatomical features are clinically relevant, their independent predictive value has not been established in the available small series [6, 11]. Operator experience, adequate catheter support, and careful procedural planning are also important when selecting the most appropriate recanalization strategy. The main anatomical and procedural factors are summarized in Table 3 [1, 6].

**Table 3:** Anatomical and procedural features relevant to crossing difficulty

Potentially more favorable features (descriptive)	Potentially challenging features (descriptive)
Short occlusion	Long occlusion
Tapered proximal stump	Blunt/absent stump
Mild calcification	Severe circumferential calcification
Minimal tortuosity	Severe tortuosity
Good distal target vessel	Poor distal target vessel
No vertebral involvement	Vertebral origin involvement
Procedural and operator-related considerations (not validated predictors)	
Experienced operator	Limited experience

These characteristics are descriptive and are not validated independent predictors of technical failure

Although advances in guidewire technology and preprocedural imaging anticipated in the earlier study have become part of contemporary practice, their impact on technical success and long-term patency has not been established in comparative SA CTO studies. Table 4 summarizes the practical advantages of these and other technological developments, together with the limitations of the available evidence [1, 5, 6].

**Table 4:** Clinical evidence and limits of inference for selected technologies

Technological development	Procedural role	Evidence base	What remains uncertain
Advanced CTA	Assessment of lesion length, proximal cap, calcification, and vertebral origin	CTA-based anatomical assessment is reported in subclavian CTO series and guidance [5, 6]	No controlled CTO-specific comparison of technical or clinical outcomes
Dedicated CTO guidewires	Additional crossing options for resistant lesions	Subclavian CTO device use described in [6]; ostial morphology/crossing experience in eight patients [11]; coronary device studies [8, 9] are indirect	Benefit of a particular guidewire over conventional equipment remains unproven
Modern crossing strategies	Alternative route when the initial crossing direction fails	Combined access in the original cohort [1] and later CTO reports [6, 10]	Historical, nonrandomized technique; its independent effect on outcomes is uncertain
Contemporary stents	Anatomically appropriate ostial placement and luminal scaffolding	Mixed subclavian/innominate cohorts [2–4]; covered-stent bailout with vertebral-origin compromise [6]	No CTO-only comparison demonstrating lower restenosis or better patency
IVUS guidance	Vessel sizing and assessment of stent expansion	Small uncontrolled subclavian obstruction cohort, mainly stenoses [12]	CTO-specific improvement in safety or durability has not been established
Overall evolution	Greater choice of imaging and crossing tools	Small CTO-only cohorts with different populations and follow-up [1, 6, 10, 14, 15]	Differences between eras cannot be attributed to devices alone

*Abbreviations*: *CTA* computed tomography angiography, *CTO* chronic total occlusion, *IVUS* intravascular ultrasound. Zhang et al. included a lesion described as subtotal; their morphology categories are exploratory, not validated CTO predictors

## Current technical challenges and future directions

Despite advances in endovascular technology, certain SA CTOs remain particularly challenging. Long, heavily calcified occlusions, a flush or poorly defined proximal cap, marked tortuosity, limited distal reconstitution, and involvement of the vertebral artery origin may make recanalization technically difficult or unsafe. Although these anatomical features influence procedural planning, their independent predictive value for technical failure has not been established [6, 11].

In selected cases, changing the crossing direction or using combined access may provide a safer approach. However, further wire manipulation should be avoided when the risk of dissection, perforation, or compromised vertebral artery flow becomes unacceptable. For symptomatic patients who still require revascularization, carotid-subclavian bypass and subclavian-carotid transposition remain important alternatives, depending on lesion anatomy, available inflow, and operative risk [5, 7].

Future developments should focus on safer recanalization strategies for these complex lesions and better identification of patients in whom EVT is unlikely to succeed.

Calcium-modification techniques may provide additional options after successful guidewire crossing of heavily calcified SA CTO, particularly when conventional balloon dilatation fails to achieve adequate expansion. Intravascular lithotripsy (IVL) has been described in a small series of calcified supra-aortic stenoses and subtotal occlusions [16] and in a recent report of severe radiation-associated SA disease with balloon underexpansion [17]. Orbital atherectomy has also been reported in an isolated case of SA CTO [18]. These reports demonstrate technical feasibility in selected lesions, but do not establish improved CTO crossing success, procedural safety, or long-term patency. Potential distal embolization and arterial injury, particularly close to the vertebral artery origin, remain important considerations [13, 18].

Prospective multicenter studies with standardized reporting of technical success, procedural complications, and long-term patency are needed to establish whether newer technologies improve clinical outcomes [2, 6, 7].

## What has actually changed in clinical practice?

The main practical change over the past 15 years is the wider range of equipment available for EVT of SA CTO. Dedicated CTO guidewires and support catheters provide additional options for complex lesions, while contemporary CTA allows more detailed assessment of occlusion morphology, calcification, and the relationship to the vertebral artery origin. However, the fundamental principles of recanalization remain largely unchanged, including adequate catheter support, controlled guidewire manipulation, confirmation of the wire position, and appropriate stent placement [1, 5, 6, 11].

Despite these technical advances, procedural safety remains an important consideration. The availability of more specialized devices does not eliminate the risks of arterial injury or neurological complications, particularly during difficult recanalization attempts.

In the original 2012 cohort of 56 patients, the overall complication rate was 7.1% [1]. Sakai et al. reported four procedure-related events during 29 procedures, including two asymptomatic stent migrations, one hemianopsia, and one transient ischemic attack. Niu et al. described a dissection extending to the aortic arch that required covered-stent implantation, with subsequent compromise of flow at the vertebral artery origin [6, 10]. In contrast, Çakar et al. reported no periprocedural complications in their series of 16 patients [15].

Late complications and the need for reintervention must also be considered. Sakai et al. reported three target-lesion reinterventions related to subacute thrombosis, in-stent restenosis, or stent-edge dissection. Niu et al. reported three cases of restenosis, whereas Çakar et al. observed one asymptomatic restenosis during 2-year follow-up [6, 10, 15]. Although these studies are not directly comparable because of differences in patient populations and follow-up, their findings highlight the importance of assessing both procedural safety and long-term outcomes when evaluating newer technologies (Table 5).

**Table 5:** Selected CTO-only and mixed cohorts: technical success and follow-up outcomes

Study	Population	Technical success	Patency (as reported)	Follow-up
CTO-only cohorts (some include other supra-aortic vessels)				
Sakai et al. [10]	26 patients/28 occlusions; subclavian and innominate arteries; mixed etiologies (including aortitis)	24/28 first attempt; 25/28 after a second procedure	Reported secondary patency 100% after three target-lesion reinterventions; this does not represent initial success in all 28 attempted lesions	Angiographic follow-up at 6 months; total follow-up duration not specified
Babić et al. [1]	56 patients/56 CTOs	46/56 (82.1%)	Primary patency 97.9% at 1 year; 82.7% at 3 years	Mean 40 ± 26 months (range 2–125)
Liu et al. [14]	67 endovascular CTO patients; 21 additional surgical comparators	52/67 (77.6%)	Primary patency 98.2% at 1 year; 94.3% at 2 years; 92.1% at 3 years after treatment	At 3, 6, 12 months and annually
Çakar et al. [15]	16 left subclavian CTO patients	15/16 (93.8%)	93.3% patency at 2 years in successfully treated cohort	Follow-up through 2 years
Niu et al. [6]	23 CTO patients	21/23 (91.3%)	Estimated primary 74.6% and secondary 78.8% at 5 years	Median 36 months (range 6–84)
Mixed stenosis/occlusion or mixed-vessel cohorts				
De Vries et al. [2]	110 patients; 90 stenoses/20 occlusions	102/110 (93%) overall; 7 occlusions not recanalized; 1 procedure stopped after ischemic stroke	5-year primary clinical patency 89% for mixed cohort; not CTO-only	Long-term estimates through 5 years
Sixt et al. [3]	107 patients/108 interventions; 78 stenoses/30 occlusions	26/30 (87%) for occlusions	1-year primary patency 88% for overall analyzed cohort; not CTO-only	One-year estimate in 97 patients eligible for follow-up
Patel et al. [4]	170 patients/177 vessels; 156 stenoses/21 occlusions; includes innominate artery	19/21 (90.5%) for occlusions	Primary patency 83%, secondary 96% at last follow-up in mixed cohort	Follow-up available for 151/170 patients; mean 35.2 ± 30.8 months

*Abbreviation*: *CTO* chronic total occlusion. Technical success is reported for attempted lesions or procedures, as defined by each study; patency generally concerns successfully treated patients or lesions. Results are not pooled. Sakai includes innominate occlusions and mixed etiologies; Liu patency refers to the endovascular cohort

### Illustrative historical procedural case (March 2008)

**Fig. 1:**
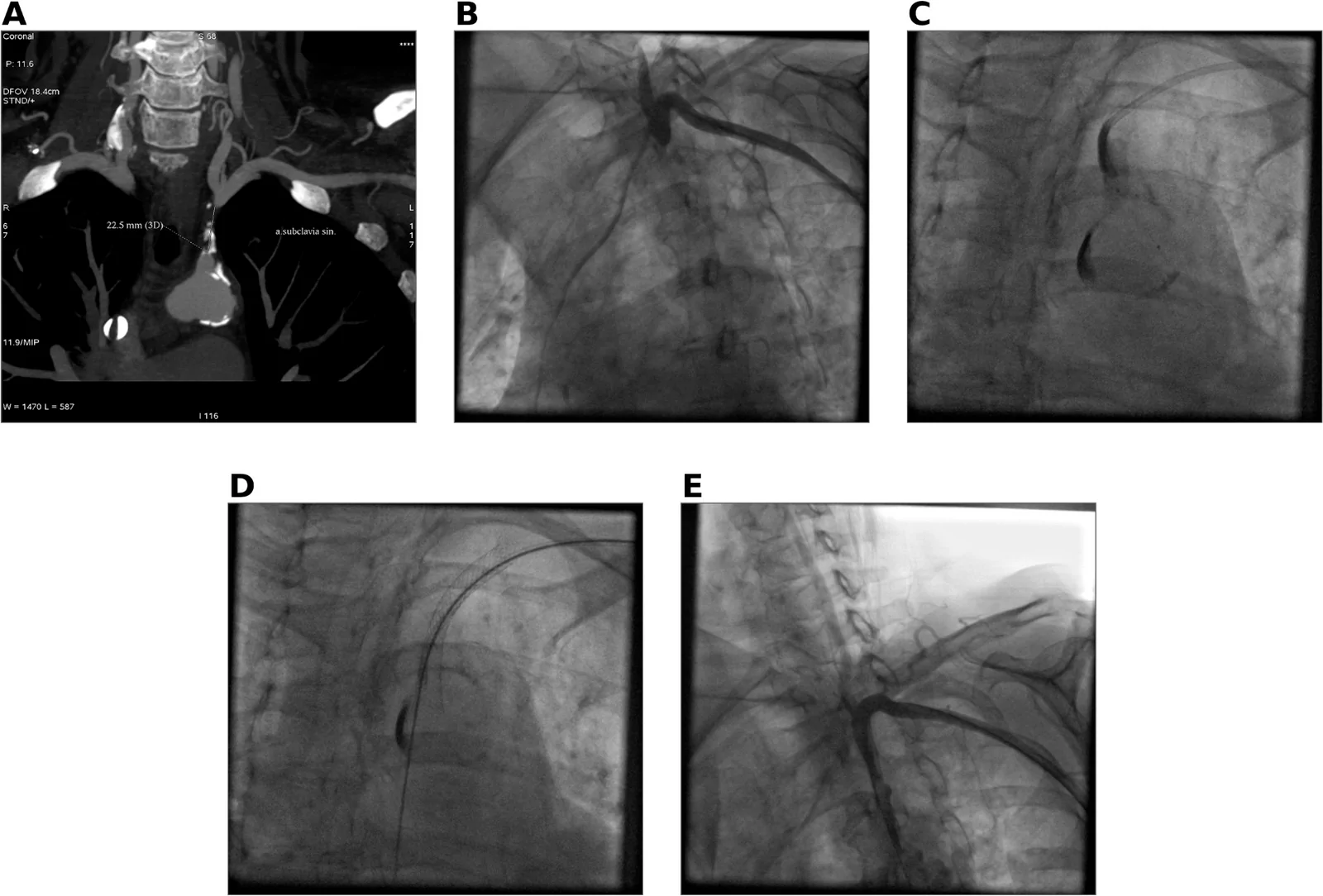
Historical endovascular recanalization of left subclavian artery chronic total occlusion (March 2008). **A** Coronal maximum-intensity projection reconstruction of preprocedural CT angiography showing a 22.5-mm-long occlusion of the proximal left subclavian artery. **B** Initial angiogram obtained via left brachial artery access, confirming complete occlusion of the proximal left subclavian artery. **C** Fluoroscopic view during recanalization using a 0.014-inch Miracle 3 guidewire supported by a 2.0 × 20-mm Maverick OTW balloon catheter. **D** Fluoroscopic image showing endovascular device positioning across the recanalized segment following guidewire passage. **E** Completion angiography after placement of an 8.0 × 40-mm Wallstent, demonstrating restored left subclavian artery patency and antegrade flow

An endovascular procedure performed in March 2008 illustrates the use of coronary-derived crossing equipment for a chronic total occlusion of the left subclavian artery (Fig. 1). Preprocedural CT angiography identified a 22.5-mm-long occlusion of the proximal left subclavian artery. Initial angiography obtained via left brachial artery access confirmed complete occlusion. Lesion crossing was achieved using a 0.014-inch Miracle 3 guidewire (Asahi Intecc, Japan), supported by a 2.0 × 20-mm Maverick over-the-wire (OTW) balloon catheter (Boston Scientific, USA). Following recanalization, an 8.0 × 40-mm Wallstent (Boston Scientific, USA) was deployed. Completion angiography demonstrated restoration of patency and antegrade flow. The procedure illustrates the combination of coronary crossing instruments with a peripheral self-expanding stent rather than an exclusively coronary device-based approach; the example is illustrative and does not establish comparative clinical benefit.

### Illustrative contemporary procedural case (2025)

**Fig. 2:**
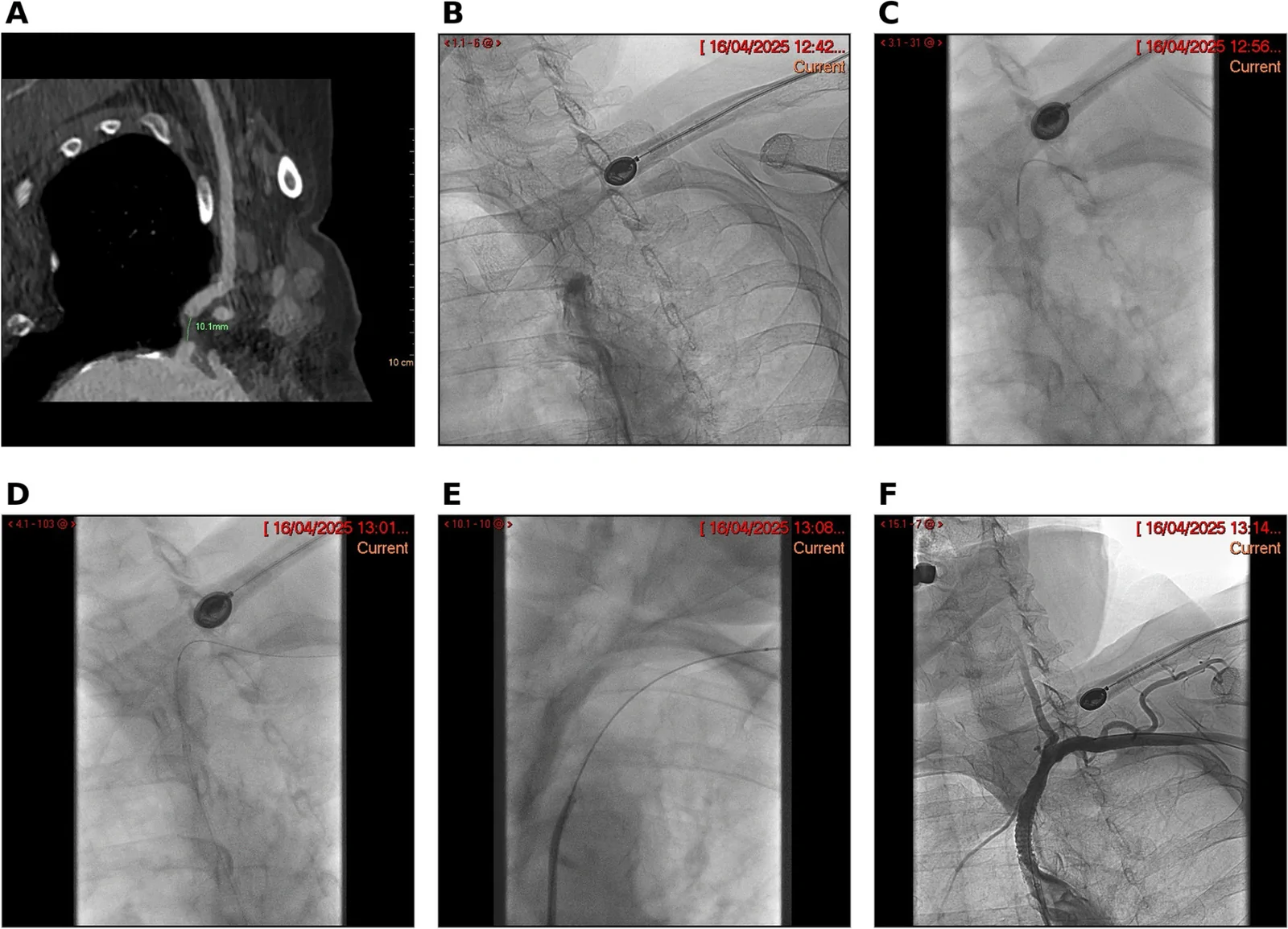
Contemporary endovascular recanalization of left subclavian artery chronic total occlusion (April 2025). **A** Preprocedural computed tomography angiography (CTA) with curved multiplanar reconstruction demonstrating a short-segment occlusion of the proximal left subclavian artery, measuring approximately 10 mm in length. **B** Initial angiography via right common femoral artery access confirming complete occlusion of the left subclavian artery. **C** Crossing of the occlusion using a 0.018-inch ASAHI Halberd guidewire supported by a NaviCross catheter. **D** Predilatation with a 5.5 × 20-mm Sterling balloon. **E** Advancement of a Destination guiding sheath over a RADIFOCUS Glidewire Advantage guidewire. **F** Completion angiography following implantation of a 7.0 × 39-mm Omnilink Elite balloon-expandable stent, demonstrating restored vessel patency and antegrade flow

An endovascular procedure performed in April 2025 illustrates the use of contemporary dedicated crossing and support devices for a left subclavian artery chronic total occlusion (Fig. 2). Preprocedural CT angiography with curved multiplanar reconstruction demonstrated a short-segment proximal occlusion measuring approximately 10 mm. Following angiography via right common femoral artery access, the occlusion was crossed using a 0.018-inch ASAHI Halberd guidewire supported by a NaviCross catheter. Predilatation was performed with a 5.5 × 20-mm Sterling balloon. A Destination guiding sheath was then advanced over a RADIFOCUS Glidewire Advantage guidewire, and a 7.0 × 39-mm Omnilink Elite balloon-expandable stent was implanted. Completion angiography demonstrated restoration of vessel patency and antegrade flow. This case illustrates contemporary device selection and delivery, but neither this procedure nor its comparison with the anatomically different 2008 case establishes improved technical success, safety, or long-term patency.

## Conclusions

Over the past 15 years, EVT of SA CTO has undergone considerable technological development. Advances in preprocedural imaging, dedicated CTO guidewires, support catheters, and stent technology have expanded the available treatment options, particularly for complex occlusions. Several developments anticipated in the earlier clinical series have become part of contemporary practice, allowing more detailed anatomical assessment and a wider choice of recanalization techniques [1, 5, 6].

Although technological advances have expanded the possibilities for successful recanalization, differences in lesion characteristics, patient selection, and study methodology prevent reliable quantification of their independent effects on technical success and long-term patency. The heterogeneous procedural techniques and follow-up reported in published CTO series further limit direct comparisons between treatment eras [1, 6, 14, 15].

Certain lesions, particularly long, heavily calcified occlusions, flush ostial CTOs, and those involving the vertebral artery origin, remain technically challenging. The fundamental principles of safe EVT, including careful anatomical assessment, appropriate access selection, adequate catheter support, and controlled guidewire manipulation, therefore remain essential. Adjunctive calcium-modification techniques, including IVL and atherectomy, may offer additional options for selected heavily calcified lesions after successful crossing, although their role in SA CTO requires further evaluation [16–18].

Future developments should focus not only on improving technical success but also on reducing procedural complications and improving long-term outcomes. Well-designed comparative studies with standardized definitions of technical success, patency, and complications are needed to establish whether further technological advances translate into meaningful clinical benefit for patients with SA CTO.

## Data Availability

Data sharing is not applicable to this article as no datasets were generated or analyzed during the current study.

## Abbreviations

3D: Three-dimensional
BE: Balloon-expandable
CT: Computed tomography
CTA: Computed tomography angiography
CTO: Chronic total occlusion
DSA: Digital subtraction angiography
EVT: Endovascular treatment
IVL: Intravascular lithotripsy
IVUS: Intravascular ultrasound
OTW: Over-the-wire
PTA: Percutaneous transluminal angioplasty
SA: Subclavian artery
SE: Self-expanding
